# Is the Acetabular Cup Orientation Different in Robot-Assisted and Conventional Total Hip Arthroplasty With Right-Handed Surgeons Using an Anterolateral Approach?

**DOI:** 10.7759/cureus.42335

**Published:** 2023-07-23

**Authors:** Gokhan Kursat Kara, Kayhan Turan, Osman Nurı Eroglu, Cagatay Ozturk, Erden Ertürer

**Affiliations:** 1 Orthopedics and Traumatology, Liv hospital Ulus, İstanbul, TUR; 2 Orthopedics and Traumatology, Atlas University, İstanbul, TUR; 3 Orthopedics and Traumatology, Liv Hospital Ulus, Istanbul, TUR; 4 Orthopedics and Traumatology, Istinye University, Istanbul, TUR; 5 Orthopedics and Traumatology, Istinye University, İstanbul, TUR

**Keywords:** hip surgery, mako, robot-assisted, acetabular cup inclination, acetabular cup anteversion, hip arthroplasty, ai & robotics healthcare

## Abstract

Introduction

Total hip arthroplasty (THA) is one of the most successful orthopaedic procedures. Survival rates from 90% at 10 years to 93% at 20 years have been reported in different studies. Differences in implant and patient characteristics can undoubtedly explain some of this variability observed in prosthesis durability, but the effect of surgical technique and implant orientation cannot be ignored. Therefore, many intraoperative methods (anatomic landmarks, intraoperative x-ray, fluoroscopy, navigation, and robotic surgery) have been attempted to avoid acetabular component malpositioning. Although postoperative computed tomography (CT) is accepted as the gold standard for the measurement of acetabular anteversion, it remains controversial in respect of costs and radiation exposure. The aim of this study was to examine how acetabular component orientation was affected in robotic and conventional THA operations performed by two surgeons with right-hand dominance.

Material and methods

The study included 113 primary THA operations performed on 113 patients between 2017 and 2022 in two groups: (i) robotic THA (Mako, Stryker Corporation, Kalamazoo, Michigan, United States) (55 patients) and (ii) conventional THA (58 patients). The patients comprised 51 males and 62 females. THA was performed on 54 right-side hips and 59 left-side hips. The operations were performed by two orthopaedic surgeons, each with 20 years of arthroplasty experience, on all the patients in the lateral decubitus position with an anterolateral approach. In all the cases, the orientation of the acetabular component was 40° inclination and 20° anteversion.

Difficult THA procedures (patients with developmental dysplasia of the hip (DDH), a history of hip surgery, revision THA, defect or deformity of the acetabulum, a history of scoliosis or lumbar posterior surgery, or those requiring proximal femoral osteotomy) were excluded from the study. Using the Liaw and Lewinnek methods, the acetabular component anteversion was measured on the radiographs taken in the optimal position postoperatively and the acetabular cup inclination angles were measured on the pelvis radiographs. The groups were compared using the Kolmogorov-Smirnov, Pearson Chi-square and Mann-Whitney U statistical tests. The limits were accepted as 40±5° for inclination and 20±5° for anteversion.

Results

No statistically significant difference was determined between the groups in respect of age, gender, or operated side. No statistically significant difference was determined between the optimal acetabular cup inclination angles of the robotic and conventional THA groups (p = 0.79). No statistically significant difference was determined between the optimal acetabular cup anteversion angles of the left and right conventional THA groups. Statistically significantly better results were determined in the robotic group in respect of acetabular cup anteversion (p<0,001).

Conclusion

The optimal orientation of the acetabular component is a key factor for successful THA. Otherwise, revision surgery is inevitable for reasons such as instability, impingement, or increased wear. The results of this study demonstrated that robotic surgery was superior to the conventional method in the placement of the acetabular component in the desired orientation.

## Introduction

Total hip arthroplasty (THA) is one of the most successful orthopedic procedures. Survival rates between 90% at 10 years and 93% at 20 years have been reported in different studies [1,2]. Differences in implant and patient characteristics can undoubtedly explain some of this variability observed in prosthesis durability, but the effect of surgical technique and implant orientation cannot be ignored [1]. Acetabular component orientation has been shown to affect the range of motion (ROM), dislocation, wear, and implant survival, but even experienced surgeons can make errors of ±15° [2].

The optimal orientation of the acetabular component is a key factor in successful THA. Otherwise, revision surgery is inevitable for reasons such as instability, impingement, and increased wear. Therefore, many intraoperative methods (manual guides, intraoperative landmarks, intraoperative x-ray or fluoroscopy, computer navigation, and robotics) have been attempted to avoid acetabular component malpositioning [3]. The method which has attracted the most interest in the last decade is robotic surgery. Robot-assisted THA is a new technology that reams the acetabulum and places the component with the help of a robotic arm on a CT-based navigation system, and has the potential to provide better component orientation than other systems [4,5]. With this method, using the preoperative CT scan and robotic arm assistance, the desired component orientation can be achieved independently of the intraoperative patient and pelvis position [1].

Just as the majority of the general population are right-hand dominant, so too are the majority of orthopedic surgeons. Very few surgeons are ambidextrous. Although there are many studies related to acetabular component orientation and probable outcomes in THA, there are extremely few studies that have examined how this is affected by hand dominance. The aim of this study was to examine how acetabular component orientation was affected in robotic and conventional THA operations performed by two surgeons with right-hand dominance.

## Materials and methods

This retrospective study was conducted at the Department of Orthopedics and Traumatology, Liv Hospital Ulus, Istanbul, Türkiye, and approved by the Ethics Committee of Istinye University (approval number: 5/2023.K-42). The study included 113 patients who underwent primary THA surgery (due to primary osteoarthritis, avascular necrosis, and femoral neck fracture) between June 2017 and December 2022. 

The study exclusion criteria were defined as: (i) revision THA; (ii) acetabular bone loss; (iii) acetabular deformity; (iv) developmental hip dysplasia; (v) BMI ≥30 kg/m^2^; (vi) preoperative or intraoperative acetabular fracture; (vii) posterolateral approach; (viii) insufficient data; (ix) insufficient postoperative radiographs; and (x) lumbar instrumentation. 

The 113 patients comprised 51 males and 62 females with a mean age of 60,16±10.24 years (range, 38-83 years) in two groups: (i) robotic THA (Mako, Stryker Corporation, Kalamazoo, Michigan, United States) (55 patients) and (ii) conventional THA (58 patients). THA was performed on 54 right-side hips and 59 left-side hips. A retrospective analysis was made of the prospectively collected medical records of the patients. All operations were performed by the same teams and the same two surgeons in this series. The anterversion and inclination values of the acetabular components were evaluated by two different senior orthopedists using the Liaw [6] and Lewinnek [7] methods.

All the operations were performed by two orthopedic surgeons with more than 20 years of professional experience. Both surgeons were right-hand dominant and both were experienced in both robotic and conventional THA. 

It was thought that the surgeon’s ability could be related to the operation side being right or left side in THA operations. As this is less affected in robotic operations, this study was planned to compare the results of two procedures and operation sides. 

Surgical technique

Robotic THA

Standardized CT scans were taken of all the patients preoperatively. The images were uploaded to the system and converted to three-dimensional (3D) form with special software.

The depth, inclination (40°), and version (20°) of the acetabular cup in the acetabulum were set preoperatively in the 3D model formed by the computer to guide the robot-assisted reaming and placement. A femoral stem of appropriate dimensions was placed in the femoral canal to determine the correct femoral neck osteotomy region to be able to restructure leg length and offset. Finally, by simulating sitting and standing femur movements, the possibilities of impingement and dislocation were investigated. Acetabular reaming and acetabular cup placement were performed in the desired manner with the assistance of the robotic arm, by introducing the patient's position and anatomy to the system intraoperatively with the help of the pelvic array, and acetabular and femoral checkpoints. 

Conventional THA

By examining the preoperative standard AP pelvic and hip x-rays of all the patients, the acetabular cup and femoral stem dimensions and orientations were determined on these radiographs with the help of templates.

All the operations were performed under general or regional anesthesia, and following the intravenous administration of prophylactic antibiotics, the patient was placed in the lateral decubitus position. After the necessary preparations, the modified Watson-Jones approach was used with a longitudinal incision over the trochanter major. Acetabular reaming and acetabular cup placement were performed with the help of the robotic arm in the robotic group, whereas in the conventional THA group, intraoperative anatomic landmarks were used. 

Postoperative radiography

Postoperatively, anteroposterior (AP) x-rays of the pelvis were taken for all the patients. Patients without standard radiographs were excluded from the study as obliquity can cause erroneous measurements.

The AP radiographs of the pelvis were obtained in the supine position at a source-to-film distance of 100 cm, with the x-ray beam centered on the superior aspect of the symphysis pubis and perpendicular to the patient. The radiographs were considered well-centered if the tip of the coccyx was centered within 2 cm of the pubic symphysis and the obturator foramen was symmetrical.

**Figure 1:**
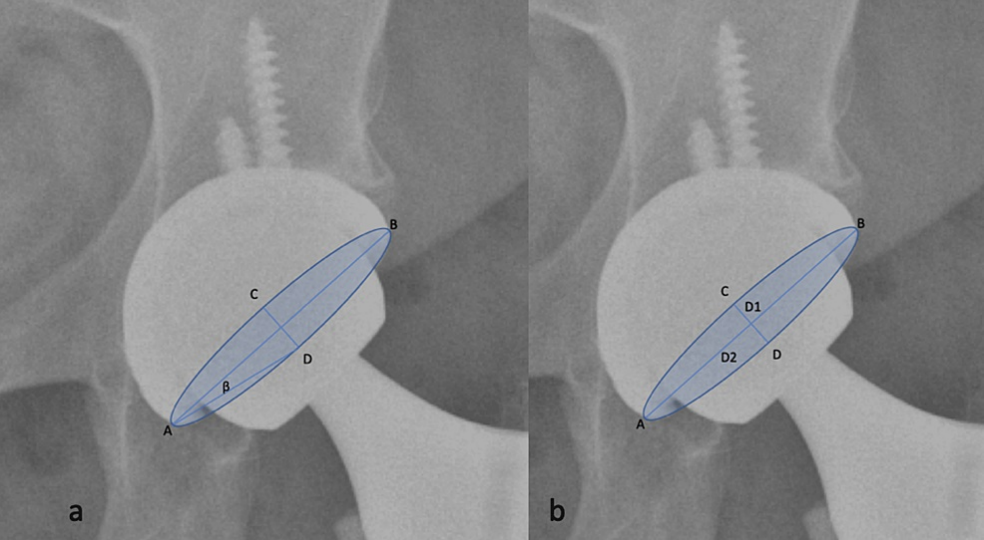
a) Liaw and b) Lewinnek methods for acetabuler anteversion measurement.

Measurement of the acetabular inclination and anteversion is shown in Figure 1.

*The Liaw Method* [6]

Version = sin −1tan β. This is a method that uses the β angle formed by the long axis of the component (AB) and the line connecting the top point of the ellipse and the end-point of the long axis.

*The Lewinnek Method* [7]

Version = arcsine (D1/D2); D1 is the distance of the short axis of an ellipse drawn perpendicular to the long axis of the acetabular component; D2 is the distance of the long axis, which is considered the maximal diameter of the implant

Acetabular Inclination

This was measured as the angle formed of the line joining the teardrops to the long axis of the acetabular component. 

Statistical analyses

The data obtained in this study were analyzed statistically using IBM SPSS Statistics for Windows, Version 25.0 (Released 2017; IBM Corp., Armonk, New York, United States). Conformity of the variables to normal distribution was examined using histograms, graphs, and the Kolmogorov-Smirnov test. Descriptive statistics are presented as mean±standard deviation, median, and minimum-maximum values. Categorical variables were compared using the Pearson Chi-square test. The Mann-Whitney U-test was used when comparing two groups of non-normally distributed (non-parametric) variables. A value of p<0.05 was accepted as statistically significant.

## Results

Between June 2017 and December 2022, 113 patients underwent hip replacement surgery with two different techniques. The robotic group included 55 patients, and the conventional THA group had 58 patients. The characteristic features were similar in both groups and both surgeons (Tables 1-2).

**Table 1:** Patients demographics for groups Chi-square tests, *Mann Whitney-U test. AVN: Avascular necrosis, Ave: Average, S: Standard deviation

		Groups				p-value
		Robotic		Conventional		
		n/Ave±SD	%/Median (min-max)	n/Ave±SD	%/Median (min-max)	
Gender	Male	27	(49,09)	24	(41,38)	0,410
	Female	28	(50,91)	34	(58,62)	
Age*		59,71±9,3	60 (40-80)	60,59±11,12	59 (38-83)	0,698
Side	Right	24	(43,64)	30	(51,72)	0,390
	Left	31	(56,36)	28	(48,28)	
Diagnosis	Osteoarthritis	39	(70,91)	42	(72,41)	0,898
	AVN	12	(21,82)	13	(22,41)	
	Femoral neck fracture	4	(7,27)	3	(5,17)	

**Table 2:** Patients demographics for surgeons Chi-square tests,  *Mann-Whitney-U test. AVN: Avascular necrosis, Ave: Average, SD: Standard deviation

		Surgeons				p-value
		1		2		
		n/Ave±SD	%/Median (min-max)	n/Ave±SD	%/Median (min-max)	
Gender	Male	27	(49,09)	24	(41,38)	0,410
	Female	28	(50,91)	34	(58,62)	
Age*		58,96±11,12	58 (38-80)	61,29±9,28	61 (40-83)	0,198
Side	Right	24	(43,64)	30	(51,72)	0,390
	Left	31	(56,36)	28	(48,28)	
Diagnosis	Osteoarthritis	36	(65,45)	45	(77,59)	0,356
	AVN	15	(27,27)	10	(17,24)	
	Femoral neck fracture	4	(7,27)	3	(5,17)	

Inclination and anteversion

The anteversion values were calculated by two senior orthopedists using the Liaw [6] and Lewinnek [7] methods. The results of these two methods were comparable (Table 3).

**Table 3:** Compatibility between Liaw and Lewinnek measurements with Intraclass Correlation Coefficient (ICC). CI: Confidence interval

	Liaw	Lewinnek
	ICC (%95 CI)	ICC (%95 CI)
Anteversion	0,991 (0,988-0,994)	0,996 (0,994-0,997)

The optimal (±5^o^) inclination and anteversion rates were compared between the two groups. The inclination success was similar (74-72%). However, in the robotic group, the rate of patients within the optimal zone for anteversion was significantly higher at 89% and it was statistically significant (p<0,001) (Table 4).

**Table 4:** Optimal inclination and anteversion distributions for groups

		Groups				p-value
		Robotic		Conventional		
		n	%	n	%	
Optimal inclination (40^o^±5^o^)	Out	14	(25,45)	16	(27,59)	0,798
	In	41	(74,55)	42	(72,41)	
Optimal anteversion (20^o^±5^o^)	Out	6	(10,91)	26	(44,83)	<0,001
	In	49	(89,09)	32	(55,17)	

Operation side

When the success rates were compared between right-side and left-side operations, no significant difference was determined in the robotic and conventional THA groups (Tables 5-6).

**Table 5:** Optimal inclination and anteversion distributions for right and left THA in conventional group. THA: Total hip arthroplasty

Conventional THA group		Side				p-side
		Right		Left		
		n	%	n	%	
Optimal inclination (40^o^±5^o^)	Out	9	(30,00)	7	(25,00)	0,670
	In	21	(70,00)	21	(75,00)	
Optimal anteversion (20^o^±5^o^)	Out	15	(50,00)	11	(39,29)	0,412
	In	15	(50,00)	17	(60,71)	

**Table 6:** Optimal inclination and anteversion distributions for right and left THA in the robotic group THA: Total hip arthroplasty

Robotic THA group		Side				p-side
		Right		Left		
		n	%	n	%	
Optimal inclination (40^o^±5^o^)	Out	4	16,67	10	32,26	0,188
	In	20	83,33	21	67,74	
Optimal anteversion (20^o^±5^o^)	Out	3	12,50	3	9,68	0,739
	In	21	87,50	28	90,32	

## Discussion

Many factors, including patient characteristics, surgical technique, and implant properties, have the potential to affect the short- and long-term results of THA. Optimal component orientation can be controlled by the surgeon and is a factor that plays a major role in preventing complications such as dislocation, rapid wear, weak biomechanics, limb length discrepancy, and revision surgery. Currently, hip instability and mechanical loosening account for more than 40% of revision hip arthroplasties, and both are directly related to component position [4].

The ideal orientation of the acetabular component was defined as anteversion 15°±10° and inclination as 40°±10° by Lewinnek et al. [7]. However, this safe zone was defined in respect of postoperative dislocation and there was later shown to be a link between an inclination of >45° and rapid wear [8]. Therefore, Callanan et al. redefined the safe zone in 2011 as anteversion 5°-25° and inclination 30°-45° [9]. In a 2013 study of 1549 THA cases, Barrack et al. reported that ideal inclination (30°-45°) and anteversion (5°-25°) could only be achieved in 37.7% [10], and Callanan et al. determined this rate as 47% in 1750 patients [9]. 

Although surgeons aim to obtain optimal component orientation, many studies have shown that this is not always possible. There is evidence that there is ±15° variability in cup orientations, even when performed by experienced surgeons. Even though ±10° is generally thought to be acceptable, new evidence has suggested that ±15° is necessary to reduce dislocation rates and there should be a margin as narrow as ±5° to optimize clinical results [11]. Therefore, the inclination and anteversion limits aimed for in the current study were defined as 45°±5° and 20°±5°, respectively. 

Although postoperative CT is accepted as the gold standard in the measurement of acetabular anteversion, it is controversial in respect of costs and radiation exposure [12,13]. Therefore, over the years several formulas have been defined with which acetabular anteversion measurements can be made on postoperative radiographs. The methods described by Liaw et al. [6] and Lewinnek et l. [7] for anteversion measurement on direct radiographs have been proven to be successful for both intraobserver and interobserver agreement and in comparative studies with CT [12-15].

In the lateral decubitus position, with either an anterolateral or posterolateral approach, right-side acetabulum reaming and component placement are more comfortable for the surgeon standing behind the patient using the right hand dominant, but it makes a difference in the left hip. In a study by Song et al., in which acetabular component orientations were compared in 498 patients who underwent bilateral THA in the same session, there was no statistically significant difference between the right and left hip acetabular component inclinations but they found that the left hip acetabular component anteversions were statistically significantly higher and more often outside the target zone [16].

In a similar study in 2019, Crawford et al. compared the acetabular component position differences between right and left hips for a right-hand dominant surgeon [17]. The results of that study showed that right-side hips had significantly lower abduction and less combined Lewinnek outliers using the direct anterior approach, and right-side hips had significantly higher anteversion and Lewinnek anteversion outliers when the posterolateral approach was used.

In our study, there was no statistically significant difference between the right and left acetabular component orientation with the conventional method. We think that this is due to the fact that both of the surgeons included in our study had more than 20 years of experience in total hip replacement surgery.

This study has several limitations. The first is the small sample size. Secondly, both of the surgeons enrolled in this retrospective study were right-handed and had rich experience in joint replacement. In our study, we used direct radiographs for the measurement of acetabular cup anteversion. Although postoperative CT is accepted as the gold standard in the measurement of acetabular anteversion, it is controversial in respect of costs and radiation exposure. In addition, all operations were performed with the anterolateral approach and in the lateral decubitus position. In our future studies, we plan to include left-handed, less experienced surgeons, and THA surgeries performed with a different approach.

## Conclusions

Robotic assistance can lead to improved accuracy and precision in component positioning. The robotic system provides real-time feedback and guidance to the surgeon, helping them achieve the desired alignment based on preoperative planning. This can potentially reduce errors associated with manual techniques, such as malpositioning or outliers. In our study where we compared acetabular orientation in robotic and conventional THA patients, we found that robot-assisted acetabular cup placement is superior in terms of acetabular anteversion.
